# Crystal structure of 3-(9*H*-carbazol-9-yl)-*N*′-[(*E*)-4-chloro­benzyl­idene]propano­hydrazide

**DOI:** 10.1107/S2056989015020770

**Published:** 2015-11-14

**Authors:** Mehmet Akkurt, Jerry P. Jasinski, Shaaban K. Mohamed, Talaat I. El-Emary, Mustafa R. Albayati

**Affiliations:** a Department of Physics, Faculty of Sciences, Erciyes University, 38039 Kayseri, Turkey; b Department of Chemistry, Keene State College, 229 Main Street, Keene, NH 03435-2001, USA; c Chemistry and Environmental Division, Manchester Metropolitan University, Manchester M1 5GD, England; d Chemistry Department, Faculty of Science, Minia University, 61519 El-Minia, Egypt; e Department of Chemistry, Faculty of Science, Assiut University, 71515 Assiut, Egypt; f Kirkuk University, College of Science, Department of Chemistry, Kirkuk, Iraq

**Keywords:** crystal structure, the carbazole ring system, bio-active mol­ecules, hydrogen bonding

## Abstract

In the title compound, C_22_H_18_ClN_3_O, the carbazole ring system is essentially planar (r.m.s deviation = 0.003 Å), and makes a dihedral angle of 9.01 (8)° with the plane of the chloro­phenyl ring. In the crystal, neighbouring mol­ecules are linked into centrosymmetric *R*_2_^2^(8) dimers by pairs of N—H⋯O inter­actions and into a three-dimensional network by C—H⋯π inter­actions. The dimers are arranged into layers parallel to (010).

## Related literature   

For synthesis and pharmacuetical studies of carbazole containing compounds, see: Hewlins *et al.* (1984 ▸); Kansal & Potier (1986 ▸); Haider *et al.* (1998 ▸); Hirata *et al.* (1999 ▸); Chowdhury *et al.* (1978 ▸); Sakano *et al.* (1980 ▸); Pindur (1990 ▸); Knölker & Reddy (2002 ▸); Martin & Prasad (2006 ▸); Saturnino *et al.* (2003 ▸).
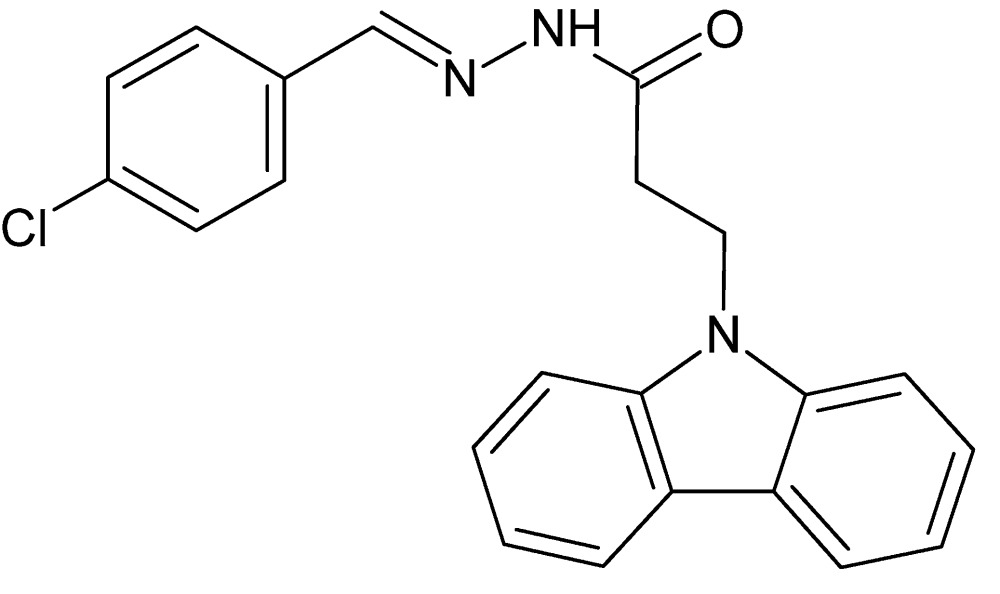


## Experimental   

### Crystal data  

C_22_H_18_ClN_3_O
*M* *_r_* = 375.84
Monoclinic, 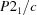

*a* = 16.0126 (7) Å
*b* = 7.4316 (3) Å
*c* = 16.1654 (9) Å
β = 94.607 (4)°
*V* = 1917.46 (16) Å^3^
*Z* = 4
Mo *K*α radiation
μ = 0.22 mm^−1^
*T* = 293 K
0.42 × 0.36 × 0.08 mm

### Data collection  

Agilent Xcalibur Eos Gemini diffractometer
Absorption correction: multi-scan (*CrysAlis PRO*; Agilent, 2014 ▸) *T*_min_ = 0.847, *T*_max_ = 1.000
12277 measured reflections
6312 independent reflections
3066 reflections with *I* > 2σ(*I*)
*R*_int_ = 0.023

### Refinement  

*R*[*F* ^2^ > 2σ(*F* ^2^)] = 0.058
*wR*(*F* ^2^) = 0.167
*S* = 1.02
6312 reflections
244 parameters
H-atom parameters constrained
Δρ_max_ = 0.20 e Å^−3^
Δρ_min_ = −0.24 e Å^−3^

### 

Data collection: *CrysAlis PRO* (Agilent, 2014 ▸); cell refinement: *CrysAlis PRO*; data reduction: *CrysAlis PRO*; program(s) used to solve structure: *SIR92* (Altomare *et al.*, 1999 ▸); program(s) used to refine structure: *SHELXL2014* (Sheldrick, 2015 ▸); molecular graphics: *ORTEP-3 for Windows* (Farrugia, 2012 ▸); software used to prepare material for publication: *PLATON* (Spek, 2009 ▸).

## Supplementary Material

Crystal structure: contains datablock(s) global, I. DOI: 10.1107/S2056989015020770/qm2112sup1.cif

Structure factors: contains datablock(s) I. DOI: 10.1107/S2056989015020770/qm2112Isup2.hkl

Supporting information file. DOI: 10.1107/S2056989015020770/qm2112Isup3.cml

**Fig. 1.**
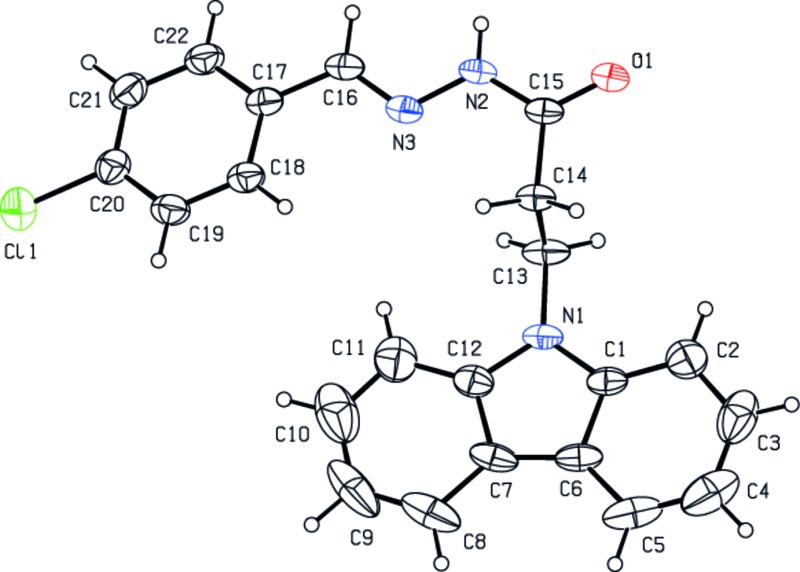
View of the title compound with the atom numbering scheme. Displacement ellipsoids for non-H atoms are drawn at the 30% probability level.

**Fig. 2.**
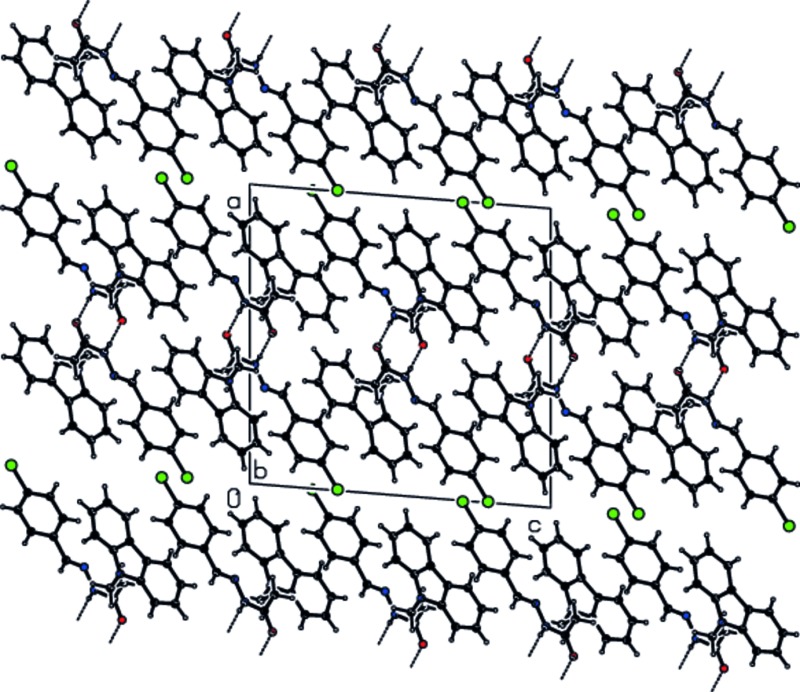
View of the dimers formed by N—H···O hydrogen bonds down the b axis.

. DOI: 10.1107/S2056989015020770/qm2112fig1.tif
View of the title compound with the atom numbering scheme. Displacement ellipsoids for non-H atoms are drawn at the 30% probability level.

b . DOI: 10.1107/S2056989015020770/qm2112fig2.tif
View of the dimers formed by N—H⋯O hydrogen bonds down the *b* axis.

CCDC reference: 1434700

Additional supporting information:  crystallographic information; 3D view; checkCIF report

## Figures and Tables

**Table 1:** Hydrogen-bond geometry (Å, °) *Cg*2, *Cg*3 and *Cg*4 are the centroids of the two benzene rings (C1–C6 and C7–C12) of the carbazole ring system and the chloro­phenyl ring (C17–C22), respectively.

*D*—H⋯*A*	*D*—H	H⋯*A*	*D*⋯*A*	*D*—H⋯*A*
N2—H2*N*⋯O1^i^	0.81	2.08	2.8952 (19)	175
C5—H5⋯*Cg*4^ii^	0.93	2.81	3.696 (3)	160
C21—H21⋯*Cg*3^iii^	0.93	2.97	3.858 (3)	160
C22—H22⋯*Cg*2^iii^	0.93	2.79	3.699 (2)	166

Symmetry codes: (i) 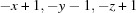; (ii) 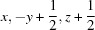; (iii) 
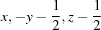
.

## supplementary crystallographic information

### S1. Comment

Carbazole scaffold compunds are well known for their pharmacological activities. The syntheses of carbazole derivatives in connection with the search for newer physiologically activities have been recognized in many reports (Hewlins *et al.*, 1984; Kansal & Potier 1986; Haider *et al.*, 1998; Hirata *et al.*, 1999). Carbazomycin A and carbazomycin B have been found to be useful antibacterial and antifungal agents (Chowdhury *et al.*, 1978; Sakano *et al.*, 1980). In addition pyridocarbazoles show marked anticancer and anti-HIV activities (Pindur, 1990; Knölker & Reddy, 2002; Martin & Prasad 2006; Saturnino *et al.*, 2003). Based on such facts we report in this study the synthesis and crystal structure of the title compound.

As shown in Fig. 1, the carbazole ring system (N1/C1–C12) of the title compound is essentially planar (r.m.s deviation = 0.003 Å), and makes a dihedral angle of 9.01 (8)° with the plane of the chlorophenyl ring (C17–C22). The bond lengths and angles are within normal ranges and are similar to those reported earlier for similar compounds.

In the crystal, two molecules are associated through a pair of N—H···O intermolecular hydrogen bonds, forming a centrosymmetric dimer with *R*_2_^2^(8) ring motifs (Table 1), into layers parallel to (010) (Fig. 2). The dimers are connected by C—H···π interactions, forming a three-dimensional network.

### S2. Experimental

A mixture of 1.5 mmol (380 mg) of 3-(9*H*-carbazol-9-yl)propanehydrazide and 1.5 mmol (261 mg) of 4-chlorobenzaldehyde was heated in 10 ml of absolute ethanol and 3 ml of acetic acid catalyst. The reaction was monitored by TLC till completion after 3 h. The product which deposited on cooling, was collected, dried under vacuum and recrystallized from dioxan to give orange plates in 78% yield.

### S3. Refinement

All H atoms were placed in calculated positions with N—H = 0.81 and C—H = 0.93 - 0.97 Å, and refined as riding with *U*_iso_(H) = 1.2*U*_eq_(C, N).

### Crystal data

**Table:**

C_22_H_18_ClN_3_O	*F*(000) = 784
*M**_r_* = 375.84	*D*_x_ = 1.302 Mg m^−^^3^
Monoclinic, *P*2_1_/*c*	Mo *K*α radiation, λ = 0.71073 Å
Hall symbol: -P 2ybc	Cell parameters from 2141 reflections
*a* = 16.0126 (7) Å	θ = 3.7–30.7°
*b* = 7.4316 (3) Å	µ = 0.22 mm^−^^1^
*c* = 16.1654 (9) Å	*T* = 293 K
β = 94.607 (4)°	Plate, orange
*V* = 1917.46 (16) Å^3^	0.42 × 0.36 × 0.08 mm
*Z* = 4	

### Data collection

**Table:**

Agilent Xcalibur Eos Gemini diffractometer	6312 independent reflections
Radiation source: Enhance (Mo) X-ray Source	3066 reflections with *I* > 2σ(*I*)
Graphite monochromator	*R*_int_ = 0.023
Detector resolution: 16.0416 pixels mm^-1^	θ_max_ = 32.8°, θ_min_ = 3.0°
ω scans	*h* = −21→24
Absorption correction: multi-scan (*CrysAlis PRO*; Agilent, 2014)	*k* = −5→10
*T*_min_ = 0.847, *T*_max_ = 1.000	*l* = −23→19
12277 measured reflections	

### Refinement

**Table:**

Refinement on *F*^2^	0 restraints
Least-squares matrix: full	Hydrogen site location: mixed
*R*[*F*^2^ > 2σ(*F*^2^)] = 0.058	H-atom parameters constrained
*wR*(*F*^2^) = 0.167	*w* = 1/[σ^2^(*F*_o_^2^) + (0.0569*P*)^2^ + 0.350*P*] where *P* = (*F*_o_^2^ + 2*F*_c_^2^)/3
*S* = 1.02	(Δ/σ)_max_ < 0.001
6312 reflections	Δρ_max_ = 0.20 e Å^−^^3^
244 parameters	Δρ_min_ = −0.24 e Å^−^^3^

### Special details

**Table:**

Geometry. Bond distances, angles *etc*. have been calculated using the rounded fractional coordinates. All su's are estimated from the variances of the (full) variance-covariance matrix. The cell e.s.d.'s are taken into account in the estimation of distances, angles and torsion angles
Refinement. Refinement on *F*^2^ for ALL reflections except those flagged by the user for potential systematic errors. Weighted *R*-factors *wR* and all goodnesses of fit *S* are based on *F*^2^, conventional *R*-factors *R* are based on *F*, with *F* set to zero for negative *F*^2^. The observed criterion of *F*^2^ > σ(*F*^2^) is used only for calculating -*R*-factor-obs *etc*. and is not relevant to the choice of reflections for refinement. *R*-factors based on *F*^2^ are statistically about twice as large as those based on *F*, and *R*-factors based on ALL data will be even larger.

### Fractional atomic coordinates and isotropic or equivalent isotropic displacement parameters (Å^2^)

**Table:**

	*x*	*y*	*z*	*U*_iso_*/*U*_eq_	
Cl1	0.99666 (4)	−0.31996 (9)	0.20792 (5)	0.0942 (3)	
O1	0.50960 (9)	−0.32276 (16)	0.57559 (9)	0.0624 (5)	
N1	0.66300 (10)	0.17932 (18)	0.56956 (10)	0.0547 (5)	
N2	0.60196 (9)	−0.39049 (19)	0.48394 (9)	0.0535 (5)	
N3	0.67596 (9)	−0.35405 (19)	0.44990 (10)	0.0511 (5)	
C1	0.64514 (12)	0.2806 (2)	0.63777 (12)	0.0539 (6)	
C2	0.57672 (16)	0.2737 (3)	0.68399 (17)	0.0803 (9)	
C3	0.5780 (2)	0.3896 (4)	0.75265 (19)	0.1108 (14)	
C4	0.6440 (3)	0.5071 (4)	0.77117 (19)	0.1203 (16)	
C5	0.7099 (2)	0.5122 (3)	0.72554 (17)	0.0944 (12)	
C6	0.71287 (14)	0.3976 (2)	0.65793 (12)	0.0609 (7)	
C7	0.77393 (13)	0.3604 (3)	0.60061 (13)	0.0649 (7)	
C8	0.85479 (19)	0.4244 (4)	0.5889 (2)	0.1049 (13)	
C9	0.8972 (2)	0.3490 (6)	0.5267 (3)	0.1362 (18)	
C10	0.8626 (3)	0.2141 (6)	0.4766 (3)	0.1306 (17)	
C11	0.78534 (18)	0.1509 (4)	0.48540 (17)	0.0887 (10)	
C12	0.74149 (13)	0.2233 (2)	0.54750 (13)	0.0578 (6)	
C13	0.61339 (13)	0.0290 (2)	0.53681 (13)	0.0646 (7)	
C14	0.63465 (11)	−0.1461 (2)	0.58248 (12)	0.0522 (6)	
C15	0.57783 (12)	−0.2941 (2)	0.54829 (12)	0.0502 (6)	
C16	0.69414 (11)	−0.4544 (2)	0.39006 (12)	0.0546 (6)	
C17	0.76989 (11)	−0.4242 (2)	0.34818 (11)	0.0507 (6)	
C18	0.83316 (12)	−0.3082 (2)	0.37949 (12)	0.0567 (6)	
C19	0.90254 (12)	−0.2777 (3)	0.33665 (14)	0.0625 (7)	
C20	0.90972 (13)	−0.3622 (3)	0.26225 (14)	0.0626 (7)	
C21	0.84950 (14)	−0.4801 (3)	0.23015 (14)	0.0701 (8)	
C22	0.78026 (13)	−0.5105 (3)	0.27385 (13)	0.0647 (7)	
H2	0.53200	0.19640	0.67050	0.0960*	
H2N	0.57290	−0.47400	0.46610	0.0640*	
H3	0.53370	0.38750	0.78640	0.1330*	
H4	0.64240	0.58410	0.81640	0.1450*	
H5	0.75350	0.59220	0.73900	0.1130*	
H8	0.87900	0.51530	0.62240	0.1260*	
H9	0.95070	0.39030	0.51840	0.1640*	
H10	0.89350	0.16560	0.43560	0.1570*	
H11	0.76210	0.06110	0.45070	0.1060*	
H13A	0.62230	0.01380	0.47860	0.0780*	
H13B	0.55450	0.05590	0.54060	0.0780*	
H14A	0.69250	−0.17810	0.57610	0.0630*	
H14B	0.62810	−0.13070	0.64120	0.0630*	
H16	0.65840	−0.54830	0.37290	0.0660*	
H18	0.82840	−0.25080	0.43000	0.0680*	
H19	0.94440	−0.20000	0.35810	0.0750*	
H21	0.85520	−0.53820	0.18000	0.0840*	
H22	0.73950	−0.59110	0.25280	0.0780*	

### Atomic displacement parameters (Å^2^)

**Table:**

	*U*^11^	*U*^22^	*U*^33^	*U*^12^	*U*^13^	*U*^23^
Cl1	0.0802 (4)	0.0860 (4)	0.1213 (6)	0.0009 (3)	0.0377 (4)	−0.0096 (4)
O1	0.0627 (8)	0.0490 (7)	0.0762 (9)	−0.0175 (6)	0.0092 (7)	−0.0065 (6)
N1	0.0572 (9)	0.0354 (7)	0.0690 (10)	−0.0128 (6)	−0.0100 (7)	−0.0014 (7)
N2	0.0576 (9)	0.0383 (7)	0.0638 (10)	−0.0184 (7)	0.0009 (7)	−0.0023 (7)
N3	0.0525 (8)	0.0393 (7)	0.0605 (9)	−0.0101 (6)	−0.0020 (7)	0.0047 (7)
C1	0.0650 (12)	0.0325 (8)	0.0620 (11)	−0.0028 (8)	−0.0089 (9)	0.0074 (8)
C2	0.0819 (16)	0.0551 (12)	0.1050 (19)	0.0077 (11)	0.0151 (14)	0.0141 (13)
C3	0.157 (3)	0.0816 (19)	0.101 (2)	0.041 (2)	0.055 (2)	0.0206 (17)
C4	0.221 (4)	0.0656 (17)	0.074 (2)	0.013 (2)	0.010 (2)	−0.0023 (15)
C5	0.160 (3)	0.0472 (12)	0.0692 (16)	−0.0157 (15)	−0.0327 (17)	0.0001 (11)
C6	0.0858 (14)	0.0358 (9)	0.0567 (11)	−0.0128 (9)	−0.0216 (10)	0.0100 (8)
C7	0.0677 (12)	0.0478 (10)	0.0745 (14)	−0.0214 (9)	−0.0224 (11)	0.0250 (10)
C8	0.0827 (18)	0.093 (2)	0.133 (3)	−0.0414 (16)	−0.0290 (17)	0.0530 (19)
C9	0.0716 (19)	0.149 (3)	0.191 (4)	−0.016 (2)	0.029 (2)	0.094 (3)
C10	0.115 (3)	0.137 (3)	0.147 (3)	0.019 (2)	0.055 (2)	0.062 (3)
C11	0.101 (2)	0.0791 (16)	0.0881 (18)	0.0100 (15)	0.0215 (15)	0.0222 (14)
C12	0.0640 (12)	0.0444 (9)	0.0637 (12)	−0.0037 (9)	−0.0031 (9)	0.0148 (9)
C13	0.0723 (13)	0.0375 (9)	0.0786 (13)	−0.0143 (9)	−0.0275 (10)	0.0013 (9)
C14	0.0569 (10)	0.0389 (8)	0.0584 (11)	−0.0108 (8)	−0.0099 (8)	−0.0005 (8)
C15	0.0580 (11)	0.0334 (8)	0.0575 (11)	−0.0100 (8)	−0.0050 (8)	0.0058 (8)
C16	0.0560 (11)	0.0377 (9)	0.0682 (12)	−0.0101 (8)	−0.0073 (9)	−0.0006 (8)
C17	0.0524 (10)	0.0370 (8)	0.0609 (11)	−0.0019 (7)	−0.0073 (8)	−0.0011 (8)
C18	0.0584 (11)	0.0493 (10)	0.0611 (11)	−0.0056 (9)	−0.0033 (9)	−0.0079 (9)
C19	0.0538 (11)	0.0509 (10)	0.0811 (14)	−0.0078 (9)	−0.0043 (10)	−0.0045 (10)
C20	0.0579 (11)	0.0515 (10)	0.0783 (14)	0.0090 (9)	0.0054 (10)	−0.0043 (10)
C21	0.0703 (13)	0.0644 (13)	0.0750 (14)	0.0045 (11)	0.0019 (11)	−0.0212 (11)
C22	0.0606 (12)	0.0534 (11)	0.0781 (14)	−0.0052 (9)	−0.0060 (10)	−0.0178 (10)

### Geometric parameters (Å, º)

**Table:**

Cl1—C20	1.733 (2)	C16—C17	1.453 (3)
O1—C15	1.229 (2)	C17—C22	1.384 (3)
N1—C1	1.384 (2)	C17—C18	1.394 (2)
N1—C12	1.373 (3)	C18—C19	1.374 (3)
N1—C13	1.446 (2)	C19—C20	1.370 (3)
N2—N3	1.373 (2)	C20—C21	1.374 (3)
N2—C15	1.345 (2)	C21—C22	1.380 (3)
N3—C16	1.274 (2)	C2—H2	0.9300
C1—C2	1.376 (3)	C3—H3	0.9300
C1—C6	1.408 (3)	C4—H4	0.9300
C2—C3	1.404 (4)	C5—H5	0.9300
N2—H2N	0.8100	C8—H8	0.9300
C3—C4	1.385 (5)	C9—H9	0.9300
C4—C5	1.336 (5)	C10—H10	0.9300
C5—C6	1.389 (3)	C11—H11	0.9300
C6—C7	1.427 (3)	C13—H13A	0.9700
C7—C12	1.405 (3)	C13—H13B	0.9700
C7—C8	1.406 (4)	C14—H14A	0.9700
C8—C9	1.377 (5)	C14—H14B	0.9700
C9—C10	1.377 (6)	C16—H16	0.9300
C10—C11	1.342 (6)	C18—H18	0.9300
C11—C12	1.380 (3)	C19—H19	0.9300
C13—C14	1.521 (2)	C21—H21	0.9300
C14—C15	1.504 (2)	C22—H22	0.9300
			
C1—N1—C12	109.24 (15)	C19—C20—C21	121.2 (2)
C1—N1—C13	124.70 (16)	C20—C21—C22	118.7 (2)
C12—N1—C13	125.17 (16)	C17—C22—C21	121.73 (19)
N3—N2—C15	121.05 (14)	C1—C2—H2	122.00
N2—N3—C16	116.50 (14)	C3—C2—H2	122.00
N1—C1—C2	129.57 (18)	C2—C3—H3	119.00
N1—C1—C6	108.41 (16)	C4—C3—H3	119.00
C2—C1—C6	121.98 (18)	C3—C4—H4	119.00
C1—C2—C3	116.4 (2)	C5—C4—H4	119.00
N3—N2—H2N	120.00	C4—C5—H5	120.00
C15—N2—H2N	119.00	C6—C5—H5	120.00
C2—C3—C4	121.4 (3)	C7—C8—H8	121.00
C3—C4—C5	121.4 (3)	C9—C8—H8	121.00
C4—C5—C6	119.7 (3)	C8—C9—H9	119.00
C5—C6—C7	134.3 (2)	C10—C9—H9	119.00
C1—C6—C7	106.58 (16)	C9—C10—H10	119.00
C1—C6—C5	119.1 (2)	C11—C10—H10	119.00
C6—C7—C8	135.1 (2)	C10—C11—H11	121.00
C6—C7—C12	107.36 (18)	C12—C11—H11	121.00
C8—C7—C12	117.5 (2)	N1—C13—H13A	109.00
C7—C8—C9	118.3 (3)	N1—C13—H13B	109.00
C8—C9—C10	121.8 (3)	C14—C13—H13A	109.00
C9—C10—C11	121.6 (4)	C14—C13—H13B	109.00
C10—C11—C12	117.9 (3)	H13A—C13—H13B	108.00
N1—C12—C7	108.37 (17)	C13—C14—H14A	110.00
N1—C12—C11	128.76 (19)	C13—C14—H14B	110.00
C7—C12—C11	122.9 (2)	C15—C14—H14A	110.00
N1—C13—C14	112.84 (16)	C15—C14—H14B	110.00
C13—C14—C15	110.00 (15)	H14A—C14—H14B	108.00
N2—C15—C14	118.09 (16)	N3—C16—H16	120.00
O1—C15—C14	121.57 (16)	C17—C16—H16	120.00
O1—C15—N2	120.28 (16)	C17—C18—H18	120.00
N3—C16—C17	120.91 (15)	C19—C18—H18	120.00
C18—C17—C22	117.87 (17)	C18—C19—H19	120.00
C16—C17—C18	122.40 (16)	C20—C19—H19	120.00
C16—C17—C22	119.72 (16)	C20—C21—H21	121.00
C17—C18—C19	120.82 (18)	C22—C21—H21	121.00
C18—C19—C20	119.68 (19)	C17—C22—H22	119.00
Cl1—C20—C21	119.48 (17)	C21—C22—H22	119.00
Cl1—C20—C19	119.30 (16)		
			
C1—N1—C12—C7	−2.1 (2)	C5—C6—C7—C8	−0.5 (4)
C12—N1—C1—C2	−175.4 (2)	C6—C7—C12—N1	1.2 (2)
C13—N1—C1—C2	−5.7 (3)	C6—C7—C12—C11	−178.4 (2)
C12—N1—C1—C6	2.3 (2)	C6—C7—C8—C9	177.5 (3)
C13—N1—C1—C6	171.93 (16)	C12—C7—C8—C9	−0.2 (4)
C12—N1—C13—C14	84.8 (2)	C8—C7—C12—N1	179.5 (2)
C1—N1—C13—C14	−83.2 (2)	C8—C7—C12—C11	−0.1 (3)
C13—N1—C12—C7	−171.72 (17)	C7—C8—C9—C10	−0.1 (6)
C1—N1—C12—C11	177.4 (2)	C8—C9—C10—C11	0.8 (7)
C13—N1—C12—C11	7.8 (3)	C9—C10—C11—C12	−1.1 (6)
C15—N2—N3—C16	178.89 (16)	C10—C11—C12—C7	0.7 (4)
N3—N2—C15—C14	−0.5 (2)	C10—C11—C12—N1	−178.7 (3)
N3—N2—C15—O1	176.58 (16)	N1—C13—C14—C15	177.03 (16)
N2—N3—C16—C17	178.32 (15)	C13—C14—C15—O1	−88.0 (2)
N1—C1—C6—C5	−179.18 (18)	C13—C14—C15—N2	89.05 (19)
N1—C1—C6—C7	−1.5 (2)	N3—C16—C17—C18	11.7 (3)
C6—C1—C2—C3	−0.1 (3)	N3—C16—C17—C22	−167.34 (18)
N1—C1—C2—C3	177.3 (2)	C16—C17—C18—C19	−177.55 (17)
C2—C1—C6—C7	176.36 (19)	C22—C17—C18—C19	1.5 (3)
C2—C1—C6—C5	−1.3 (3)	C16—C17—C22—C21	177.23 (19)
C1—C2—C3—C4	1.5 (4)	C18—C17—C22—C21	−1.9 (3)
C2—C3—C4—C5	−1.5 (5)	C17—C18—C19—C20	0.0 (3)
C3—C4—C5—C6	0.0 (5)	C18—C19—C20—Cl1	178.96 (16)
C4—C5—C6—C7	−175.5 (3)	C18—C19—C20—C21	−1.3 (3)
C4—C5—C6—C1	1.4 (4)	Cl1—C20—C21—C22	−179.29 (17)
C5—C6—C7—C12	177.4 (2)	C19—C20—C21—C22	1.0 (3)
C1—C6—C7—C12	0.2 (2)	C20—C21—C22—C17	0.7 (3)
C1—C6—C7—C8	−177.6 (3)		

### Hydrogen-bond geometry (Å, º)

Cg2, Cg3 and Cg4 are the centroids of the two benzene rings (C1–C6 and C7–C12) of the carbazole ring system and the chlorophenyl ring (C17–C22), respectively.

**Table:**

*D*—H···*A*	*D*—H	H···*A*	*D*···*A*	*D*—H···*A*
N2—H2*N*···O1^i^	0.81	2.08	2.8952 (19)	175
C14—H14*A*···N3	0.97	2.42	2.765 (2)	100
C5—H5···*Cg*4^ii^	0.93	2.81	3.696 (3)	160
C21—H21···*Cg*3^iii^	0.93	2.97	3.858 (3)	160
C22—H22···*Cg*2^iii^	0.93	2.79	3.699 (2)	166

Symmetry codes: (i) −*x*+1, −*y*−1, −*z*+1; (ii) *x*, −*y*+1/2, *z*+1/2; (iii) *x*, −*y*−1/2, *z*−1/2.
