# Supplementary figures and images for: Crystal structure of 3-(9H-carbazol-9-yl)-N′-[(E)-4-chloro­benzyl­idene]propano­hydrazide
Source: Acta Crystallogr E Crystallogr Commun. 2015 Nov 14;71(Pt 12):o937–8. doi: 10.1107/S2056989015020770 (PMC4719890; doi:10.1107/S2056989015020770)

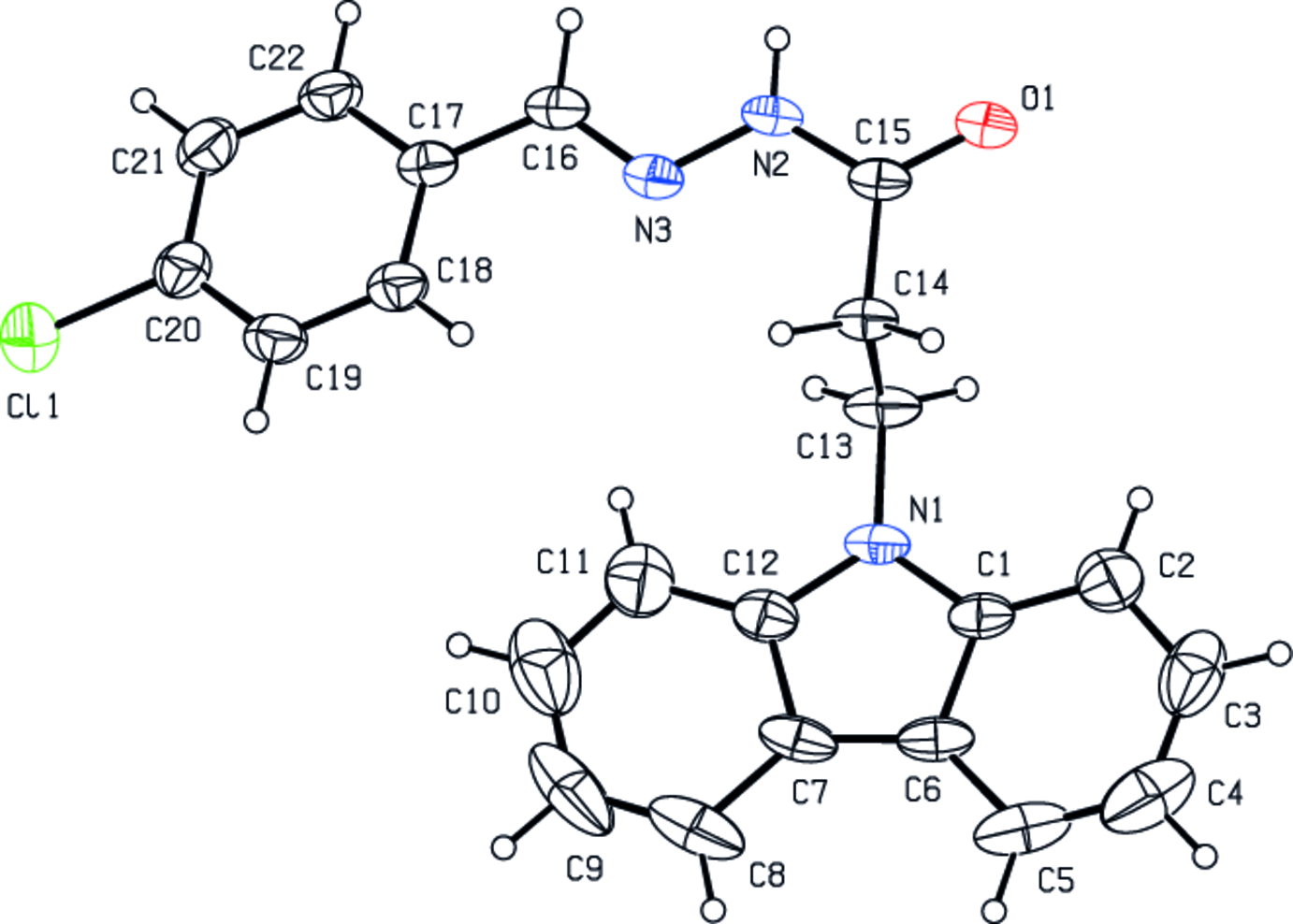

Supplement: Supplementary file 4 [file e-71-0o937-fig1.tif]

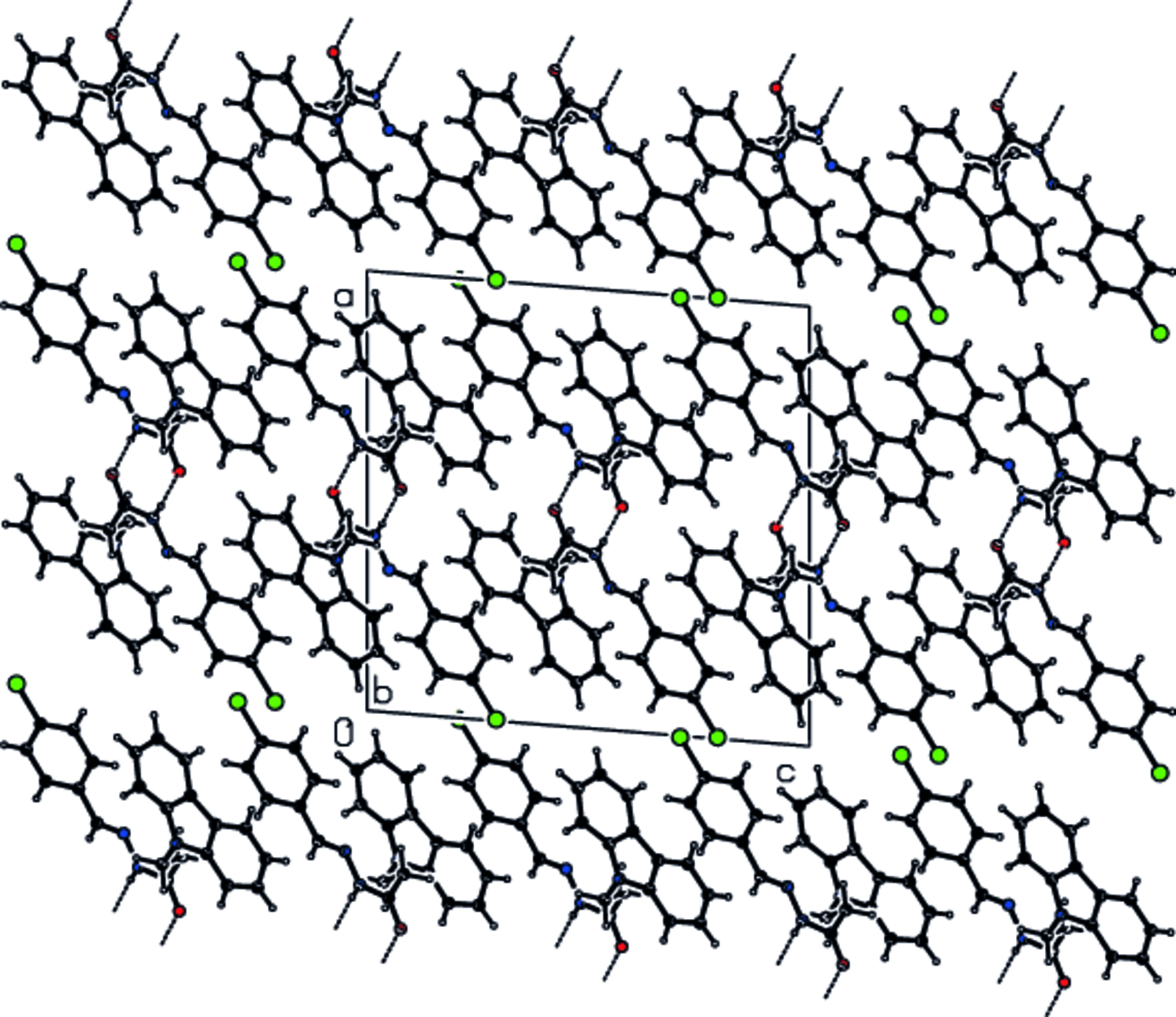

Supplement: Supplementary file 5 [file e-71-0o937-fig2.tif]
